# Expression of the Pupal Determinant *broad* during Metamorphic and Neotenic Development of the Strepsipteran *Xenos vesparum* Rossi

**DOI:** 10.1371/journal.pone.0093614

**Published:** 2014-04-07

**Authors:** Deniz F. Erezyilmaz, Alex Hayward, Yan Huang, Jordi Paps, Zoltan Acs, Juan A. Delgado, Francisco Collantes, Jeyaraney Kathirithamby

**Affiliations:** 1 Department of Biochemistry and Cell Biology, Stony Brook University, Stony Brook, New York, United States of America; 2 Science for Life Laboratory, Department of Medical Biochemistry and Microbiology, Uppsala University, Uppsala Biomedical Centre, Uppsala, Sweden; 3 Department of Zoology, University of Oxford, Oxford, United Kingdom; 4 Kaposvar University, Faculty of Animal Science, Kaposvar, Hungary; 5 Departamento de Zoologia, Facultad de Biologia, Universidad de Murcia, Murcia, Spain; Natural Resources Canada, Canada

## Abstract

Derived members of the endoparasitic order Strepsiptera have acquired an extreme form of sexual dimorphism whereby males undergo metamorphosis and exist as free-living adults while females remain larviform, reaching sexual maturity within their hosts. Expression of the transcription factor, *broad* (*br*) has been shown to be required for pupal development in insects in which both sexes progress through metamorphosis. A surge of *br* expression appears in the last larval instar, as the epidermis begins pupal development. Here we ask if *br* is also up-regulated in the last larval instar of male *Xenos vesparum* Rossi (Stylopidae), and whether such expression is lost in neotenic larviform females. We clone three isoforms of *br* from *X. vesparum (Xv’br)*, and show that they share greatest similarity to the Z1, Z3 and Z4 isoforms of other insect species. By monitoring *Xv*’*br* expression throughout development, we detect elevated levels of total *br* expression and the *Xv’Z1*, *Xv’Z3*, and *Xv’Z4* isoforms in the last larval instar of males, but not females. By focusing on *Xv*’*br* expression in individual samples, we show that the levels of *Xv’BTB and Xv’Z3* in the last larval instar of males are bimodal, with some males expressing 3X greater levels of *Xv’br* than fourth instar femlaes. Taken together, these data suggest that neoteny (and endoparasitism) in females of Strepsiptera Stylopidia could be linked to the suppression of pupal determination. Our work identifies a difference in metamorphic gene expression that is associated with neoteny, and thus provides insights into the relationship between metamorphic and neotenic development.

## Introduction

In terms of both diversity and abundance, the insects are one of the most successful animal classes [1]. Many groups within the insects are distinguished by sharp life history transitions that occur at molts. Such life history switches occur between two fully differentiated states that are tailored to exploit different resources, to cope with environmental changes, or to subdivide labor [2]. The most common life history polymorphism among the insects is complete metamorphosis, which is the central trait that defines the group Holometabola, which includes butterflies, beetles, bees and flies. In the Holometabola, metamorphosis between the larval form and the winged, sexually mature adult form occurs over two molts, the molt to the pupal stage and the final molt to the adult stage. This strategy allows the larval stages to specialize in feeding and growth, while the adult form specializes in dispersal and reproduction. Much research has focused on the regulation of metamorphosis in holometabolous insects. Little is known, however, of whether the mechanisms used to regulate metamorphosis are also used to regulate other types of life history polymorphisms.

**Figure 1:**
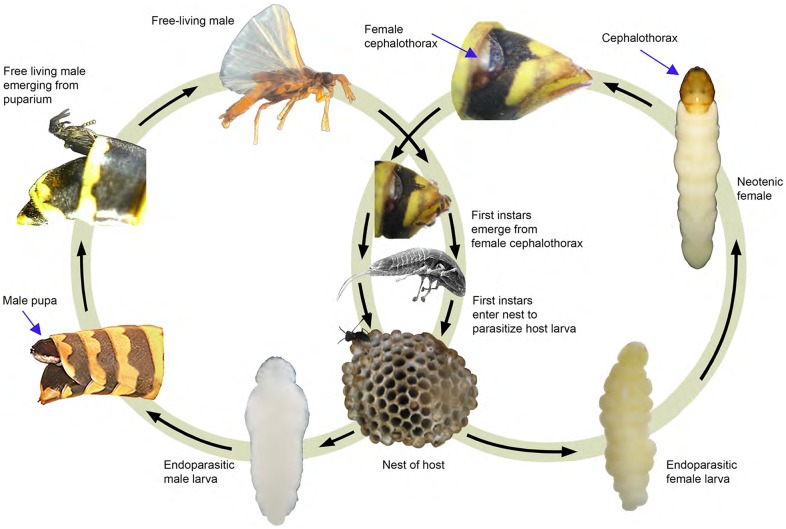
Life cycle of *Xenos vesparum* within its paper wasp host, *Polistes dominula*. A free-living male (top left) mates with the endoparasitic neotenic female via her extruded cephalothorax, which protrudes through the host cuticle (top right). The female produces first instar larvae that exit through the brood canal in the extruded cephalothorax. The free-living 1^st^ instar larvae (center) seek a host within paper wasp nests. Upon entering a host, the 1^st^ instars molt to apodous endoparasitic second instar larvae with a soft cuticle. The second instar larvae molt two additional times. Male 4^th^ instar larvae (bottom left) molt to form a pupa, which extrudes from the abdomen of the host cuticle. At the end of pupal and adult development, the free-living male emerges from the ecdysed cuticles and from its host. Female 4^th^ instar larvae (bottom right) do not undergo additional molts, but instead develop a cephalothorax, which is extruded through the host cuticle for the purposes of mating and release of 1^st^ larvae.

Some of the most dramatic switches in insect form are found in the holometabolous order Strepsiptera (Figure 1). All strepsipterans are endoparasites, and switches between forms occur at transitions between the free-living and endoparasitic stages. Female Strepsiptera produce their young by hemocoelous vivipary, and produce 1^st^ instar larvae that are specialized for host seeking that have rudimentary eyes, thoracic limbs, and a sclerotized cuticle. Upon finding a host, the first switch in differentiated form occurs as the 1^st^ instar larva molts to an endoparasitic, apodous larva with a soft cuticle [3]. In the early branching order of Strepsiptera, the Mengenillidae, both male and female larvae emerge from the host to form puparia, and eventually produce free-living male and wingless neotenic female adults. However in the more derived Stylopiformia, females exhibit an extreme form of neoteny, and remain endoparasitic within the host, existing as a “bag of eggs” [4], [5]. In *Xenos vesparum* Rossi, a parasite of paper wasps that is a member of Stylopidia, male 4^th^ instar larvae molt to the pupal and adult stages. Upon completion of adult development, the fully differentiated free-living male emerges from the endoparasitic puparium [4],[6]. *X. vesparum* females do not form a puparium after the 4^th^ instar. Instead they remain larviform, and lack eyes, mouthparts, antennae, wings and external genitalia as adults. During the final larval instar, the head and anterior thoracic region of the female is extruded through the host cuticle, and sclerotizes to form the chephalothorax. This structure facilitates mating and the exodus of 1^st^ instar larvae [4]–[6]. Therefore, two modifications of typical holometabolous insect life history are found in Strepsiptera: 1) the switch between the free-living 1^st^ instar larva and the apodous endoparasitic larval form that lives within the host, and 2) the loss of a switch between the apodous, larval stage and the pupal stage in female Stylopidia.

Two hormones, juvenile hormone (JH) and ecdysone, regulate transitions between instars and progression through metamorphosis. The timing of molts is triggered by the steroid hormone ecdysone, which is released in temporally regulated pulses. The presence of JH during the larval stages prevents progression to the pupal stage, while the presence of JH at the pupal stage prevents progression to the adult stage [7]. A number of functional studies have established that JH-dependent switches exert their effect through expression of the JH-effector, *broad (br)* [8]–[15]. Expression of high levels of *br* is restricted by JH and ecdysone to the larva- pupa transition in holometabolous insects [16]–[18]. Loss of *br* expression at this stage results in larval-adult hybrids in the flour beetle *Tribolium castaneum* (Herbst) [11]–[13]. in the silkmoth *Bombyx mori* (L.) [9] and in the lacewing *Chrysopa perla*,(L.) a fairly primitive holometabolous insect [11]. *br* null mutants in *Drosophila melanogaster* Meigen fail to enter metamorphosis altogether [8].

The *br* locus is complex, and produces multiple transcripts through alternative splicing of an N-terminal Broad-Tramtrack-Bric-a-brac (BTB)-containing core region to one of several C2H2 zinc finger-containing C-terminal sequences. The *Broad* gene is best characterized in *D. melanogaster*, where alternative splicing produces many different transcripts, with all splice products containing one of four zinc finger types Z1–Z4 [19],[20]. The four zinc finger isoforms have varied expression levels and temporal profiles in different tissues ([20]–[22]. Each isoform in *D. melanogaster* binds DNA and regulates metamorphosis-specific expression of target genes [22]–[24].

Here we propose that the appearance of neotenic females in Strepsiptera is linked to the modification of pathways that underlie pupal determination. To test this hypothesis, we isolate *broad* sequences from the male and female *X. vesparum*, which exhibits extreme sexual dimorphism. We examine the expression of total *Xv’br* and of three *br* isoforms, and ask: 1) whether *Xv*’*br* expression is up-regulated in the last larval instar as it is in other holometabolous insects, and 2) whether this up-regulation is missing in endoparasitic neotenic females.

## Methods

### Strepsiptera Collection

*X. vesparum* specimens used for cloning and gene expression experiments were collected from one of two locations: 1) Ajka, Veszprem county, Hungary, (47° 2′ 29.29″N, 17° 32′ 57.38″E). Unstylopized and stylopized wasps were transported from Hungary to England (Oxford) speedily in spacious, airy plastic boxes with food and blotting paper. In the laboratory at Oxford, paper wasp larvae of the species *Polistes dominula* (Christ) were infected individually in the nest using 1^st^ instars that emerged from neotenic female *X. vesparum.* Records of infection date for each colony were kept so host wasps and developing *X. vesparum* specimens could be sacrificed at varying intervals post-infection to provide a developmental time series. After dissection of stylopized wasp larvae, *X. vesparum* specimens were stored in RNAlater (Life Technologies, Norwalk, CT) at −80°C**.** 2) A second set of *X. vesparum* specimens were obtained from a moderately parasitized population of *P. dominula* living in an Aleppo pine (*Pinus halepensis* Mill) Mediterranean forest, located in the central part of the Sierra Espuña Range Natural Park, Murcia province of southeastern Spain (37° 51′ 28.0″N, 1° 31′ 10.5″W). Wasps were kept alive and transported to the lab at ambient temperature. All collected exemplars of *P. dominula* were dissected in saline solution with *X. vesparum* samples preserved in absolute alcohol.

### Ethics Statement

Stylopized wasps from Ajka, Hungary were collected on privately owned land. Permission to collect was granted by the landowner. For the Spanish material, permission to collect in all the protected areas of the Murcia Region was granted by the Dirección General de Medio Ambiente of the Comunidad Autónoma de la Región de Murcia. These permits are available upon request.

### Cloning and Sequence Analysis

We used degenerate primers designed to amplify a 137 bp fragment of the conserved BTB domain [25] from genomic DNA. The amplified fragment was sequenced to design additional primers for RACE as follows: 5′ RACE (29 bp): CTG CAG GCA GAG AGG ACG ACT CGG TGG GC and 3′ RACE (36 bp): GCA GCA TTA CCT CTG CTT TCG AGA ACC TAC GGG ATG. Male early 3^rd^, early 4^th^, early pupae, and mid-pupal stages were combined in a single Trizol (Life Technologies, Norwalk, CT) extraction to generate cDNA. The Clontech SMART RACE Kit (Mountain View, CA) was used to extract 5′ and 3′ regions. All PCR and RACE products were sequenced in both directions. The full-length coding sequences have been deposited in GenBank (*Xvbr’Z1* =  KJ465870, *Xvbr’Z3* =  KJ465871, *Xvbr’Z4* =  KJ465872).

### Bioinformatics

A semi-automated BLAST and HMM-based search was conducted, using as queries insect *br* sequences obtained from the online Pfam database [26] and the Pfam HMM profile for BTB (PF00651.26).

### Phylogenetic Analysis of Isoforms

Nucleotide sequences for 41 *br* isoforms originating from diverse insect species were obtained from Genbank (Table S1), and aligned together with the *X. vesparum br* sequences that were isolated in this study using MUSCLE v3.8.31 [27]. The appropriate model of sequence evolution for the 162 bp alignment was identified as GTR+G using the Akaike Information Criterion in jModelTest v0.1.1 [28]. Phylogenetic relationships were estimated using Bayesian Inference in MrBayes v3.2 [29]. The analysis was run for four million generations using the (MC)^3^ algorithm, with four simultaneous Markov chains (three heated, one cold). Prior to chain termination, the standard deviation between split frequencies was verified as being below 0.01. Bayesian posterior probabilities were estimated for each clade from the fifty per cent majority-rule consensus tree for the sampled trees (excluding burn-in) of two million generations.

For the BTB domain, the sequences were data mined from transcriptome data (Boussau et al, unpublished) using BLAST and HMMER. They were then aligned with MAFFT [30]. The resultant alignment of 115aa positions, was used to infer a gene tree with the program RAxML [31] using the most complex evolutionary model available (LG + Gamma + Invariant sites), and 1,000 bootstrap replicates. We used the Geneious software (Aukland, NZ) to graph the alignment.

### RT-PCR

To isolate RNA for RT-PCR, we used the Qiagen RNeasy Plus Kit (Valencia, CA). For most stages, we isolated RNA from individual Strepsiptera. In the case of second instar larvae, however, we combined 9 larvae in a single extraction in order to isolate measurable RNA. Only RNA samples that showed intact ribosomal RNA bands on an agarose gel were used to generate cDNA. 250 ng of RNA from two individuals were combined for each stage (except for the 2^nd^ instar samples), and used in reverse transcriptase reactions using VILO Superscript RT Mix (Life Technologies, Norwalk, CT). Control reactions to test for DNA contamination were generated by first denaturing the reverse transcriptase enzyme at 65°C. We did not detect genomic contamination in any RNA sample. We performed at least two biological replicates for each stage. For each region assayed, we subcloned and sequenced the fragments to confirm their identity. For each primer pair, we optimized reaction conditions with a dilution series from 100 ng/uL to 0.01 ng/uL to assure that our amplification was in the linear range. The primers and number of cycles used for each region of the *Broad* gene, and for 18S ribosomal cDNA are listed in Table S2.

### Quantitative Real Time PCR

The integrity of each RNA sample was checked on an agarose gel, and 500 ng of intact RNA was used in reactions with VILO Superscript RT Mix (Life Technologies, Norwalk, CT). Real Time assays were performed on a Light Cycler 480 using Roche Sybr Green Master Mix (Madison, WI). One one-hundredth of a cDNA reaction was used for each real time reaction, and three real-time reactions were performed on each sample. 18S amplification was used as a reference to normalize reactions. The Pfaffl equation [32], R =  E_target_^ΔCp (mean control - mean sample)^/E_reference_^ΔCp(mean control - mean sample)^ was used to incorporate measurements of individual samples, reaction efficiencies (E) and 18S expression.

## Results

**Figure 2:**
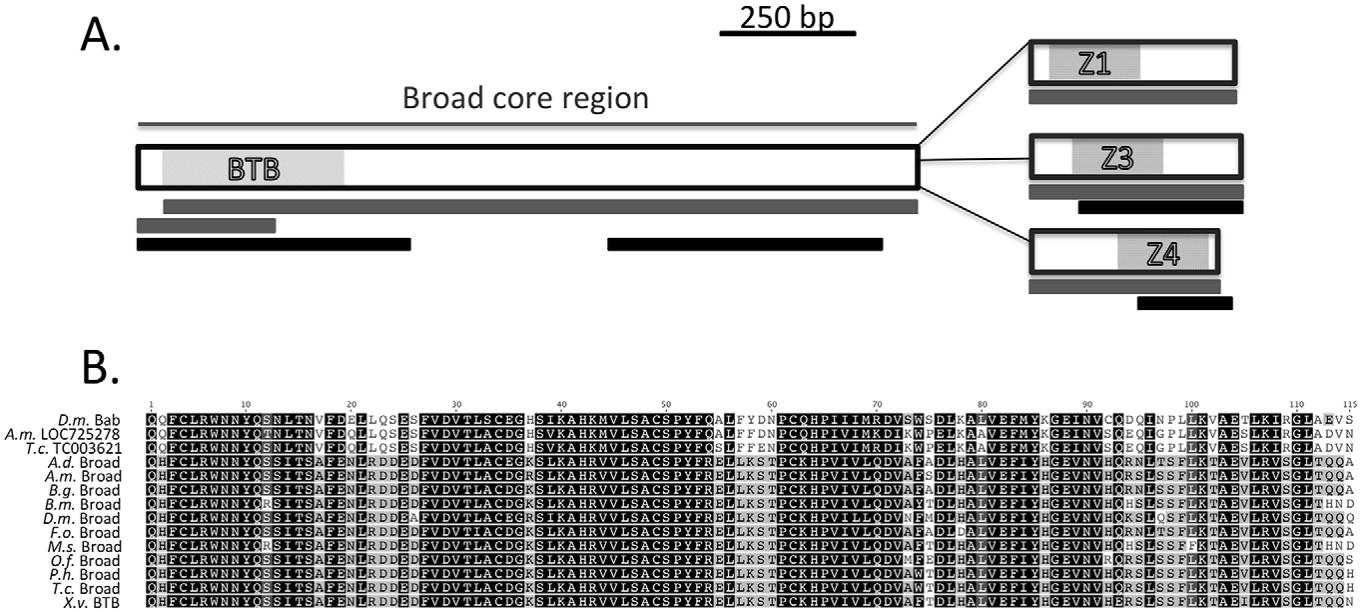
Cloning and characterization of *Xenos vesparum Broad* Isoforms. A. Black bars are derived from predicted transcriptome sequences. Clones derived from RACE are denoted with gray bars. B. A Global alignment of 115aa of the conserved BTB domain from *X. vesparum* and eight other insects for which Broad has been characterized. We used the BTB domain from the *D. melanogaster* protein, Bric-a-Brac (Bab) as an outgroup (D.m. Bab). Broad protein sequences are shown from the species *A.d. = Acheta domesticus, A.m. =  Apis melifera, H.s. = Harpegnathos saltator, B.g. = Blattella germanica, B.m. =  Bombyx mori, D.m*. = *Drosophila melanogaster, F.o. =  Frankliniella occidentalis,* M.s. = *Manduca sexta, O.f.* = *Oncopeltus fasciatus*, *P.h. =  Psacothea hilaris, T.c. = Tribolium castaneum.* The BTB domain from the broad gene of *X. vesparum* identified here =  *X.v*. BTB. We also include the BTB sequence from an uncharacterized *Apis mellifera* protein, an uncharacterized BTB (*A.m.* LOC725278), and an uncharacterized BTB domain from a *T. castaneum* protein (*T.c*. TC003621), as outgroups.

The *Broad-complex* encodes multiple transcripts that are produced through splicing a common BTB/POZ containing ‘core’ to alternate C2H2 zinc finger sequences. We isolated a 137 bp fragment of the *X. vesparum* BTB domain using degenerate primers [25], and used this region to generate primers for 5′ and 3′RACE. Three zinc finger isoforms were isolated by 3′RACE using cDNA that was derived from mixed 3^rd^ and 4^th^ instar larval and mixed pupal RNA. The amplified sequences show greatest similarity with other insect broad-complex sequences encoding the Z1, Z3 and Z4 isoforms (Figure 2). We also searched an RNA-seq library generated from *X. vesparum* females using BLAST searches (NCBI; Boussau et al, unpublished data). This analysis produced sequences that overlapped the BTB domain, core region and Z3 and Z4 zinc fingers (Figure 2). The *X. vesparum* Br BTB domain clustered with other insect Br BTB domains on a phylogenetic tree (Figure S1).

**Figure 3:**
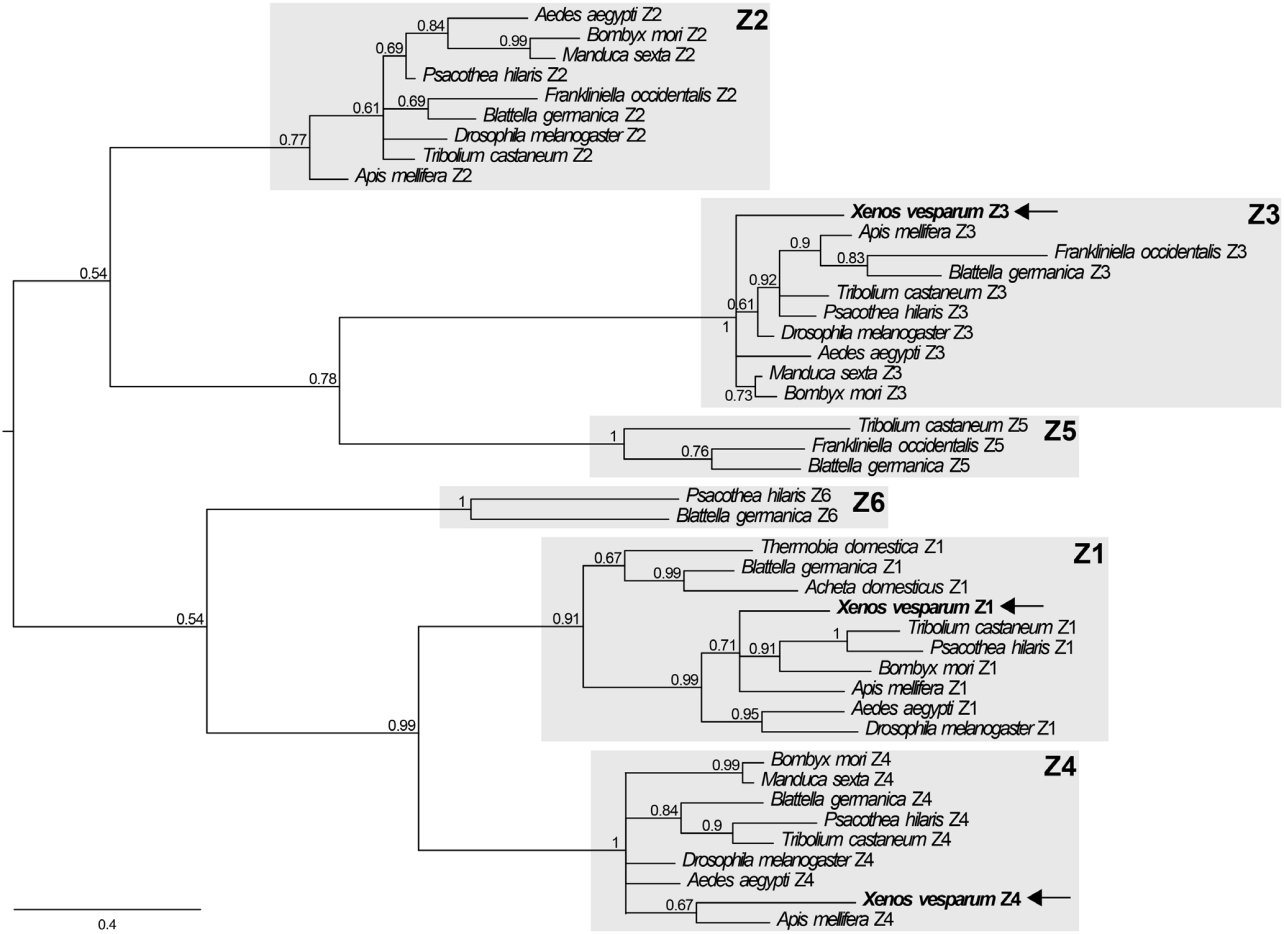
Bayesian phylogenetic tree comparing a 162 nucleotide alignment of known C2H2 zinc finger isoforms (Z1–Z6) from *X. vesparum* (in bold, and indicated by arrows) and five other insects with characterized *broad* zinc fingers. Numbers presented to the left of nodes represent posterior probability values. See SI Table 1 for corresponding accession numbers.

The results of a phylogenetic analysis of the *X. vesparum* zinc finger isoform sequences isolated in this study, together with isoform sequences from a group of holo- and hemimetabolous insects obtained from GenBank are presented in Figure 3. The analysis clearly demonstrates that the isoforms obtained from *X. vesparum* group with known insect isoforms corresponding to insect *br Z1, Z3,* and *Z4*. In *D. melanogaster*, the *Broad-Complex* produces four zinc finger isoforms. However, we were unable to isolate a Z2 isoform by searching the transcriptome of female *X. vesparum* or by RACE, despite repeated attempts. Although an additional Z5 isoform has been discovered in *T. castaneum* [11],[14], and Z5 and Z6 isoforms have been identified in *Blatella germanica* L. [33], our search did not uncover zinc finger regions that clustered with the Z5 or Z6 isoforms (Figure 3).

**Figure 4:**
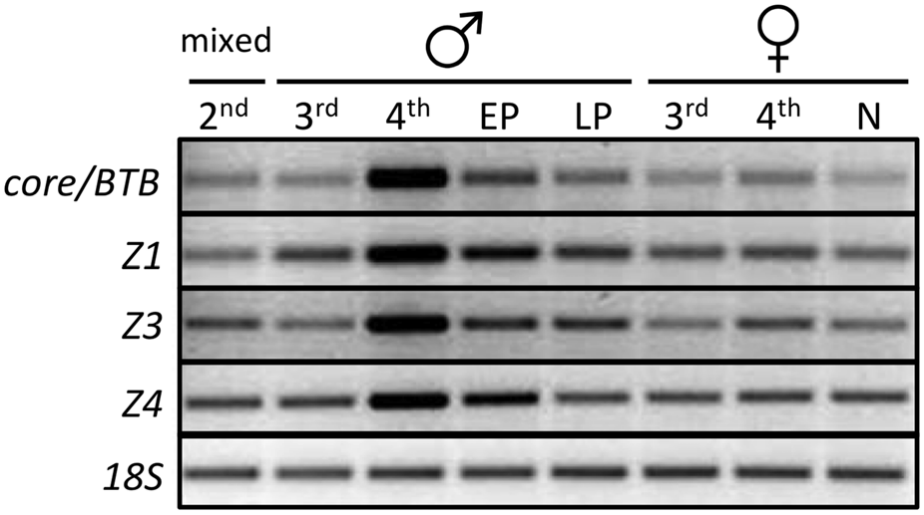
RT-PCR expression of the Broad core (core/BTB), the three zinc finger isoforms (Z1, Z3, Z4), and 18S ribosomal RNA in staged *X. vesparum*. The labels on the Y-axis denote stages. 2^nd^ = mixed male and female second instar, 3^rd^ = 3^rd^ instar, 4^th^ = 4^th^ instar, EP =  early pupa, LP = mid-late stage pupa, N =  neotenic female.

*Br* expression is up-regulated at the last larval instar of holometabolous insects as the process of metamorphosis begins [9],[11]–[14],[16],[17]. Lower levels of expression have been detected in whole animal homogenates during each larval stage. In *D. melanogaster*, low levels of *br* expression in the larva correspond to neural expression of the Z3 isoform [34], while the epidermis determines the type of cuticle generated at each instar. In contrast, an analysis of epidermal *br* expression in the moth, *M. sexta* shows that *br* is temporally restricted to the final larval instar [16],[17]. *X. vesparum* larvae pass through four larval molts. Only males enter metamorphosis, while females remain larviform. We therefore asked whether an up-regulation of *br* expression, corresponding to the surge in epidermal expression in *M. sexta*, might occur in homogenates of male *X. vesparum* 4^th^ instar larvae, but not in female 4^th^ instar larvae. The *br* BTB-containing core (*Xv’BTB*) and the three zinc finger isoforms were detected in mixed homogenates of each stage. We found a consistent increase of both *Xv’BTB* and each of the zinc fingers in the 4th instar of males that was not seen in females. The increase in *Xv’BTB*, *Xv’Z1*, *Xv’Z3* and *Xv’Z4* transcripts persisted through the first part of the male pupal stage (Figure 4). These data suggest that the surge of *br* expression seen in the last larval instar of holometabolous insects occurs in *X. vesparum* males, but not in females.

**Figure 5:**
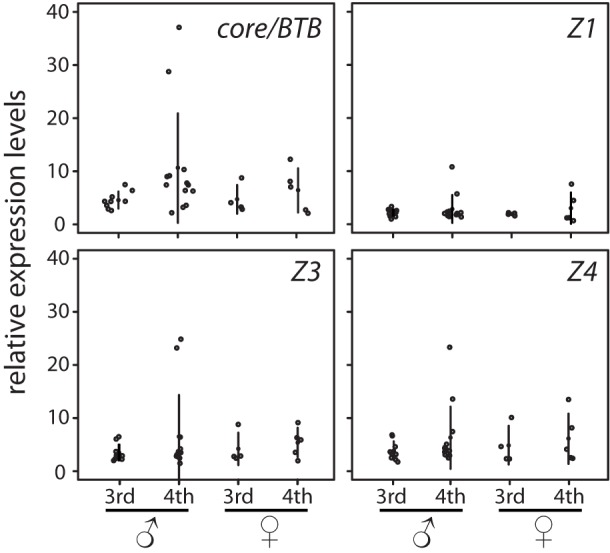
Real-Time PCR comparison of *br* gene expression of male and female 3^rd^ and 4^th^ instar larvae. RNA levels of *Xv’BTB, Xv’Z1*, *Xv’Z3* and *Xv’Z4* relative to a standard cDNA reaction were tested in nine stage 3 males, 13 stage 4 males, four 3^rd^ instar females and five 4^th^ instar females. Filled circles represent the mean relative levels; open circles indicate measurements of individual samples. The error bars show standard deviations.

A surge in expression of *br* in other metamorphosing insects is temporally restricted to the final 25–50-% of the instar [11],[12],[14],[16] by the concentration of ecdysone [17],[18]. We were unable to stage larval instars in *X. vesparum* because Strepsiptera are endoparasitic. Consequently, we cannot be certain of the exact time point during the molting cycle that RNA was collected. To address this limitation, we quantified expression of *Xv’BTB, Xv’Z1*, *Xv’Z3* and *Xv’Z4* in all 31 individual samples of 3^rd^ and 4^th^ instar male and female larvae that were available in our collections (Figure 5). We find no significant difference in the mean expression levels of *Xv’BTB, Xv’Z1*, *Xv’Z3* or *Xv’Z4* between the male 3^rd^ instar, female 3^rd^ instar, male 4^th^ instar and female 4^th^ instar (ANOVA *p* = 0.183 for *Xv’BTB*, Figure 5). By contrast, we find that the sample variance between expression levels of 4^th^ instar males and females were significantly different for the Z3 isoform (F-test, *p* = 0.028), for *br* core, *Xv’BTB* (F-test, *p* = 0.047), but not for the Z1 and Z4 isoforms (*p* = 0.340 and 0.360, respectively). The difference in sample variances that we find for *Xv’BTB* and *Xv’Z3* expression is due to a bimodal distribution in expression of *Xv’BTB* and *Xv’Z3* within the 4^th^ instar male sample (Figure 5). For *Xv’BTB* for instance, most of the 4^th^ instar males have a mean of 6.6+/−2.5 relative expression units, but two outliers in this group show expression levels that are 4- and 6-fold greater levels than average expression. Moreover, *Xv’BTB* levels in the highest-expressing 4^th^ instar males is 6.7-fold greater than the highest expressing 3^rd^ instar male, 4.2-fold greater than the highest expressing 3^rd^ instar female, and 3-fold greater than the highest expressing 4^th^ instar female (Figure 5). This pattern is shared among the three zinc finger isoforms that we analyzed, and elevated expression levels of *Xv’Z1*, *Xv’Z3* and *Xv’Z4* are found in the same two samples of 4^th^ instar males.

## Discussion

We isolated three complete *br* coding sequences from the strepsipteran *X. vesparum* that phylogenetically cluster with previously defined insect *br* sequences. Br proteins belong to a group of DNA binding proteins that possess an N-terminus BTB domain and a C terminus zinc finger domain. An analysis of the crystal structure of other BTB domains reveals that this motif forms dimers [35]. Dimerization creates one surface for interaction with DNA, and another that interacts with co-activators and co-repressors of transcription [35]–[37]. A conserved lysine, at position 5 in Figure 2B, is required for dimerization, and for protein function. This residue is conserved in *Xv*’BR, as well as all the other insect Br proteins. An aspartic acid residue at position 29 and an arginine residue at position 43 in our alignment (Figure 2B) have been shown to be crucial for recruitment of regulators of chromatin conformation, such as histone deacetylases, nuclear receptor co-receptor (N-coR) and silencing mediator of retinoic acid and thyroid hormone receptor (SMRT) [36],[37]. We find that these residues are also conserved in the *X. vesparum* Br and all other insect Br BTB domains, making it likely that the insect Br proteins also regulate downstream gene expression through dimerization and subsequent recruitment of histone modifiers of chromatin.

The *br* sequences we obtained for *X. vesparum* code for three distinct transcripts that separate with the Z1, Z3 and Z4 isoforms, grouping them with previously characterized sequences on a gene tree. We were unable to isolate a Z2 transcript from *X. vesparum*, despite repeated attempts using 3′ RACE or by BLAST searches of a *X. vesparum* transcriptome (unpublished data). At least four zinc finger-containing isoforms are present in the annotated genomes of other insect species, which each contain a Z2 isoform. Thus, a Z2 isoform may also be expressed in *X. vesparum*, but possibly not at high enough levels to be isolated in our RNA libraries made from the final larval instars, the pupal stages and adult females. Alternatively, it may be that *X. vesparum* genuinely lacks this isoform. Future research may help to elucidate if this is indeed the case, or if the Z2 is present, but at very low levels or over very short timescales.

In most holometabolous insects, *br* expression during the last half of the last larval instar is necessary for the switch between larval and pupal forms [8],[9],[11],[12],[14],[18]. Up-regulation of *br* expression during the last larval instar is determined by the hormone ecdysone, and is best shown in studies of *M. sexta* epidermis. A small elevation in ecdysone levels appears in a brief pulse midway through the final larval instar. The lower levels are not sufficient to induce a molt, but instead are sufficient to induce pupal commitment, a developmental ‘point of no return’ when the epidermal tissue switches from a larval to a pupal fate [38]–[40]. Expression of *br* is induced at pupal commitment, and its expression at this point is required for pupal development [16]–[18]. Although we were unable to stage *X. vesparum* larvae from the onset of the instar due to their endoparasitic lifestyle, we expect that within an instar where pupal commitment occurs, two levels of *br* expression would be detected; those larvae that have not yet experienced pupal commitment will retain low levels of *br,* while those larvae that have undergone pupal commitment will show high levels of br. We found that *Xv’BTB* (core) and *Xv’Z3* expression support a scenario where *Xv’br* induction occurs in the last larval instar in males (Figure 4, 5), and that elevated expression persists into the pupal stage, just as it does in other holometabolous insects (Figures 4,5). In contrast, we do not detect a similar induction in 4^th^ instar females. However, our sample size for this group was limited to 5 individuals and we cannot exclude the possibility that a larger sample size might reveal higher levels of *Xv’br* expression in neotenic females.

A comparison of the role of *br* genes in different insect groups reveals that the salient function of *br* is to permit switches between alternate forms. Throughout holometabolous insects, *br* expression is required for the larval-pupal transition [8],[9],[11],[12],[14]. In hemimetabolous insects, *br* is up-regulated at the end of each larval stage [10],[41],[15]. In the hemimetabolous milkweed bug, *Oncopeltus fasciatus* (Dallas), switches between nymphal forms, or heteromorphosis, requires *br* expression; loss of *Of’br* at any nymphal stage results in a repetition of the existing nymphal morphology [10]. This aspect of *br* function suggests that *br* is uniquely suited to regulate other types of insect polymorphisms. Our data suggests that loss of a switch in *br* expression in *X. vesparum* may underlie the emergence of neoteny in Strepsiptera.

A second example of an atypical *br* expression pattern occurs in the propupa of thrips, hemimetabolous insects that have evolved a quiescent and non-feeding ‘propupa’ and ‘pupa’ stage independently. Metamorphosis in thrips involves replacement of larval specific structures, such as mouthparts and antennae, with adult structures. The wings begin to grow outside of the cuticle during the propupal or pupal stages [42]. *br* expression in thrips is low until the onset of propupal development, then declines during the pupal stage [43]. Although the function of *br* expression has not been tested in the thrips, these data show that novel expression of *br* correlates with the appearance of a novel switch in life history progression.

Paedogenesis, the retention of juvenile traits in adults that may occur through neoteny, has evolved at least 6 times in insects [44]. However, little is known of the genetic or endocrine mechanisms that have been altered during evolution to produce neotenic development. One exception is work on the gall midge, a facultative paedomorph that produces mature ovaries during the larval stage when conditions are favorable. The rate of ovarian follicle formation in the gall midge, *Heteropeza pygmaea* Winnertz can be regulated in culture by ecdysone and JH [45]. The ecdysone receptor, (EcR) is a nuclear receptor that dimerizes with a second protein, ultraspiracle (USP), to mediate tissue-specific responses to ecdysone. In the ovaries of *D. melanogaster*, EcR/USP heterodimers appear in the last larval instar and are required for proper progression of ovarian differentiation [46]. Similarly, an increase in EcR/USP proteins is found in the final larval instar of the gall midges *H. pygmaea* and *Mycophila speyeri* (Barnes), during metamorphosis while EcR/USP proteins are upregulated early in the first larval instar during paedogenetic development [44]. These findings suggest that shifts in the timing of endocrine regulators of metamorphosis, like EcR/USP or *br*, in one tissue with respect to the entire organism may account for the development of paedogenetic development. In the case of *X. vesparum*, the metamorphosis pathway appears to have become decoupled from sexual maturation, but only in females. This may have occurred through loss of the pupal commitment ecdysone peak, by severing connections between the commitment peak and *br* expression, or through mutations in the *Xv’br* regulatory region. Our study establishes a starting point for discovery of the origin of extreme neoteny in the enigmatic order Strepsiptera, and provides a novel contribution to research into animal life-history transitions more widely.

## Supporting Information

Figure S1 **Broad BTB domain Tree.** 345 nucleotides of 11 insect Broad BTB domains are compared with BTB domains from other insect proteins. *A.d. = Acheta domesticus, A.m. = Apis melifera, B.g. = Blattella germanica, B.m. = Bombyx mori, D.m*. = *Drosophila melanogaster, F.o. = Frankliniella occidentalis, M.s*. = *Manduca sexta, O.f.* = *Oncopeltus fasciatus*, *P.s. = Psacothea hilaris, T.c. = Tribolium castaneum*. *A.m.* LOC725278 is an uncharacterized BTB-containing protein from *Apis mellifera*. *T.c*. TC003621 is an uncharacterized BTB domain from a *T. castaneum* protein, and *D.m*. Q960S0 BTB-protein-VII is an uncharacterized BTB-containing protein from *D. melanogaster* used as an outgroup. Dm Q9W0k4 is the BTB domain from the protein, Bric-a-brac.
(PDF)

Table S1 **Insect Species and Accession numbers.** Insect sequences and the GenBank Accession numbers that were used to construct the zinc finger sequence tree (Figure 3).
(PDF)

Table S2 **RT-PCR primers.** The sequences of primers used in RT-PCR and real time PCR. The number of cycles are given for RT-PCR analysis.
(PDF)
