# Supplementary figures and images for: Expression of the Pupal Determinant broad during Metamorphic and Neotenic Development of the Strepsipteran Xenos vesparum Rossi
Source: PLoS One. 2014 Apr 7;9(4):e93614. doi: 10.1371/journal.pone.0093614 (PMC3977908; doi:10.1371/journal.pone.0093614)

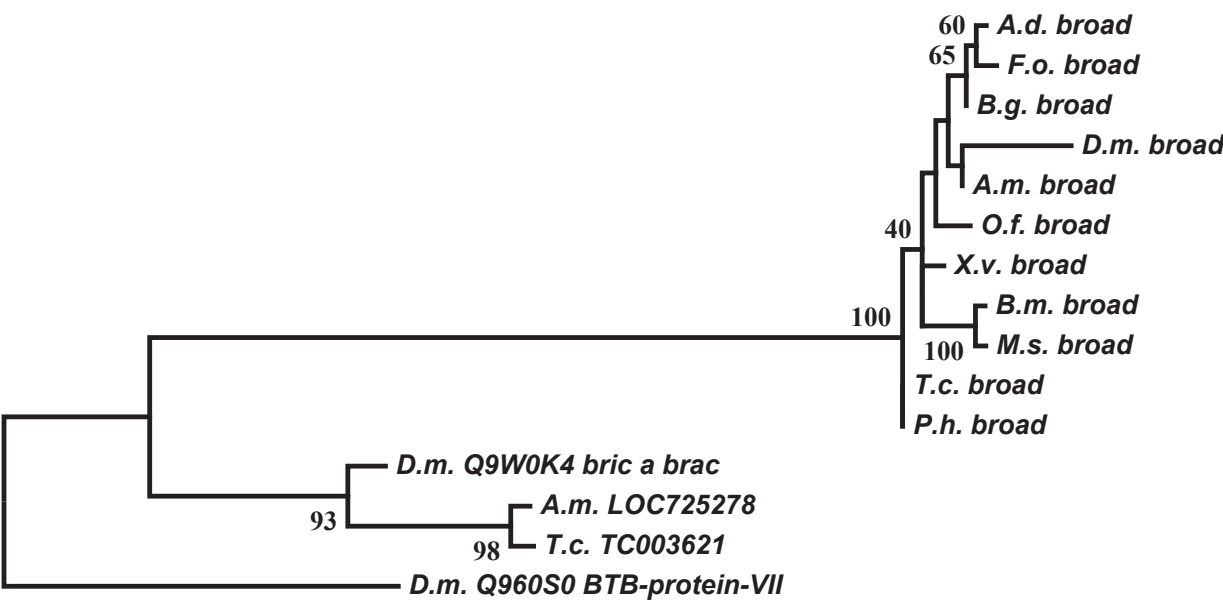

0.1

Supplement: Figure S1 — Broad BTB domain Tree. 345 nucleotides of 11 insect Broad BTB domains are compared with BTB domains from other insect proteins. A.d. = Acheta domesticus, A.m. = Apis melifera, B.g. = Blattella germanica, B.m. = Bombyx mori, D.m. = Drosophila melanogaster, F.o. = Frankliniella occidentalis, M.s. = Manduca sexta, O.f. = Oncopeltus fasciatus, P.s. = Psacothea hilaris, T.c. = Tribolium castaneum. A.m. LOC725278 is an uncharacterized BTB-containing protein from Apis mellifera. T.c. TC003621 is an uncharacterized BTB domain from a T. castaneum protein, and D.m. Q960S0 BTB-protein-VII is an uncharacterized BTB-containing protein from D. melanogaster used as an outgroup. Dm Q9W0k4 is the BTB domain from the protein, Bric-a-brac. (PDF) [file pone.0093614.s001.pdf]
