# Supplementary material for: Expression of the Pupal Determinant broad during Metamorphic and Neotenic Development of the Strepsipteran Xenos vesparum Rossi
Source: PLoS One. 2014 Apr 7;9(4):e93614. doi: 10.1371/journal.pone.0093614 (PMC3977908; doi:10.1371/journal.pone.0093614)
Supplement: Table S1 — Insect Species and Accession numbers. Insect sequences and the GenBank Accession numbers that were used to construct the zinc finger sequence tree (Figure 3). (PDF) [file pone.0093614.s002.pdf]

Species	Broad isoform	Genbank accession
Acheta domesticus	Z1	DQ176003
Aedes aegypti	Z1	AY499537
Aedes aegypti	Z2	AY499538
Aedes aegypti	Z3	AY499539
Aedes aegypti	Z4	AY499540
Apis mellifera	Z1	AB208106
Apis mellifera	Z2	AB208107
Apis mellifera	Z3	AB208108
Apis mellifera	Z4	AB207270
Blattella germanica	Z1	FN651774
Blattella germanica	Z2/3	FN651780
Blattella germanica	Z3	FN651776
Blattella germanica	Z4	FN651777
Blattella germanica	Z5	FN651778
Blattella germanica	Z6	FN651779
Bombyx mori	Z1	NM_001111334
Bombyx mori	Z2	NM_001111333
Bombyx mori	Z3	AB181201
Bombyx mori	Z4	NM_001043511
Drosophila melanogaster	Z1	X54666
Drosophila melanogaster	Z2	X54665
Drosophila melanogaster	Z3	X54664
Drosophila melanogaster	Z4	U51585
Frankliniella occidentalis	Z2	AB572569
Frankliniella occidentalis	Z3	AB572570
Frankliniella occidentalis	Z5	AB572571
Manduca sexta	Z2	AF032674
Manduca sexta	Z3	AF032675
Manduca sexta	Z4	AF032676
Psacothea hilaris	Z1	AB857715
Psacothea hilaris	Z2/Z3	AB858990
Psacothea hilaris	Z3	AB858991
Psacothea hilaris	Z4	AB858992
Psacothea hilaris	Z5/Z6	AB858993
Psacothea hilaris	Z6	AB858994
Thermobia domestica	Z1	GQ983556
Tribolium castaneum	Z1	EU200754
Tribolium castaneum	Z2	AB370884
Tribolium castaneum	Z3	EU200756
Tribolium castaneum	Z4	EU200757
Tribolium castaneum	Z5	EU200752
