# Supplementary material for: Expression of the Pupal Determinant broad during Metamorphic and Neotenic Development of the Strepsipteran Xenos vesparum Rossi
Source: PLoS One. 2014 Apr 7;9(4):e93614. doi: 10.1371/journal.pone.0093614 (PMC3977908; doi:10.1371/journal.pone.0093614)
Supplement: Table S2 — RT-PCR primers. The sequences of primers used in RT-PCR and real time PCR. The number of cycles are given for RT-PCR analysis. (PDF) [file pone.0093614.s003.pdf]

Region	Forward Primer	Reverse Primer	# Cycles
BTB Domain	GCAGCATTACCTCTGCTT	CGAAAATATGGGCTGCAG	23
Z1	TGTCAGCCATGCAATAAGAGT	GCGTAAACTATTTAAGCTACTG	26
Z3	TGTCCATATTGTCATAGGACG	GTTGTAAGCTTTTGTGAGTAGT	27
Z4	GCGGCAAAATGCTAAGCACT	GGTGATAAATACTTCGATGATTA	27
18S	TGCGGCGTATCTTTCAATTGT	CTGCCTTCCTTAGATGTGGT	16
